# The Impact of the Innovative Knowledge of Customers on Their Recommendation Intentions

**DOI:** 10.3389/fpsyg.2020.00979

**Published:** 2020-06-03

**Authors:** Fenghua Zhang, Depeng Zhang, Mengfei Lin

**Affiliations:** ^1^School of Management, Guangdong University of Technology, Guangzhou, China; ^2^School of Economics and Management, Zhuhai City Polytechnic, Zhuhai, China

**Keywords:** customer knowledge, innovative activities, cognitive fit, guidance method, recommendation intentions

## Abstract

This article provides a systematic way to examine the impact of the innovative knowledge of customers on their recommendation intentions from firm design perspective and investigates the moderating effects of guidance methods and design materials provided combining different aspects of cognitive fit, media richness, and sticky information theories. We use the EQXIU platform, conduct two experiments, and find that there are significant differences between the novice customers and expert customers in their recommendation intentions. Experts are more prone to no-template materials, whereas novices are more inclined to use modules and templates. Therefore, to inspire innovation, firms should offer personalized opportunities based on customers’ knowledge levels to enhance their experience. Firms should also design the innovative activities taking into consideration the knowledge levels of their customers. At last, limitations of this study and directions for further research are discussed.

## Introduction

Learning is a key driving factor for customers to take part in firm innovative activities, through which they not only discover how other people do things but also learn how to engage themselves. Successful completion of an innovative activity following the appropriate guidance methods (GMs) and employing materials may increase their sense of achievement and their motivation. In addition, understanding customer behavior benefits the firm by providing valuable insights to future business practices. Identifying appropriate GMs during the design of innovative activities based on customers’ type and the creation of personalized experiences and materials within the activities are crucial for firms’ survival and success.

Businesses are increasingly paying attention to customer innovation. For example, Whirlpool Corporation used mobile applications to develop new products and reduce the failure rate, whereas Adidas proposed the “MI ADIDAS” project to allow for mass customization of their products. Firms from information technology, retail, and food services have encouraged customers to offer their innovative knowledge and participate actively in the product experience and design. For instance, Xiaomi (the “Apple of China”) offers various hardware, software, and internet services based on customers’ feedback. Millet TV encourages users to share innovative knowledge through WeChat and other social network platforms and has attracted millions of participants. McDonald introduced “Create Your Taste” in China in 2015 to enable customers to unleash their burger creativity. This initiative was followed by a series of “homemade burgers” on “WeChat Moments” in Shanghai, resulting in immediate word-of-mouth (WOM) recommendations.

A core issue in customer innovation is the appropriate management of customer knowledge, which is the acquisition, sharing, shifting, efficient usage, and updating of such knowledge. Chang and Taylor (2016) argue that firms need to effectively use customer knowledge and should enhance knowledge management through customer involvement. Numerous studies have noted that a profound personal experience can clearly stimulate WOM spread from an innovative customer (Schreier et al., 2007; Agag and El-Masry, 2016). Indeed, Kumar et al. (2010) suggest that an important element of “customer engagement value” is “customer recommendation value.” However, designing marketing activities that motivate customer to participate in innovative experiences and give recommendations remains a challenge for management.

Literatures on the impact of customer knowledge on WOM recommendations are not uncommon (Brucks, 1985; Bansal and Voyer, 2000; Wangenheim and Bayón, 2004), but the customer knowledge in these studies tended to focus on *the knowledge about customers* and *the knowledge possessed by customers*, which is the knowledge mainly from ordinary customers, whereas this article focuses on *the knowledge cocreation with customers*, which is generated and delivered in the process of firm innovation activities by innovative customers. As customer innovation and the cocreation value have attracted more and more attention of academia and industry, it is very necessary to study the innovative knowledge displayed by innovative customers in the process of participating in innovation activities. Moreover, customers with higher knowledge levels are able to complete innovative tasks more efficiently and more precisely, because the innovative knowledge of customer has been considered for the new product development and innovation quality (Chang and Taylor, 2016; Taghizadeh et al., 2017). Although Fidel et al. (2015) have examined the impact of customer innovative knowledge management on performance, to the best of our knowledge, few researchers have studied how customer innovative knowledge affects WOM recommendations from the perspective of firm design for now. The firm design perspective means that in the process of how customer innovative knowledge affects WOM recommendations, the firm exerts its active effect. In order to drive customers to make better recommendations, the firm should consciously choose customers and design the key link of the activity to match the knowledge level of customers, so that they are more willing to recommend to others.

The aims of this article are to investigate the influence of the innovative knowledge of customers on their recommendation intentions, as well as the moderating effects of the different GMs and design materials provided. Our research intends to address the following three research questions:

1. How customer types (experts vs novices) influence their experience in innovative activities?
2. What are the factors that motivate each group to further promote their experience via WOM recommendations?
3. Are the level of customer innovative knowledge and the recommendation intentions indeed related?

Based on cognitive fit theory, media richness, and sticky information, we use two experiments to test our hypotheses on the role of customer innovative knowledge, GMs, and design materials provided. Our research has two main contributions. First, this article provides a systematic way of examining the influence of innovative knowledge and WOM recommendations from firm design perspective. Second, this article investigates the moderating effects of GMs and design materials provided combining different aspects of theories. The findings of this article contribute to related research on customer knowledge management and provide implications to practitioners and designers.

The remainder of this article is organized as follows: in *Literature Review*, we present the literature review related to the research question posed in our work, and we position our article within the body of the literature. In *Proposed Research Framework*, we present the proposed research framework and the hypothesis that will be tested. In *Experimental Design and Data Analysis*, we present the two-step analysis that we designed to test the hypothesis defined. *Discussions and Implications* discusses the results and makes implications. Finally, *Conclusions and Limitations* proposes the inclusions and limitations.

## Literature Review

In this section, we provide a brief literature review of the work already done in the areas of customer knowledge, knowledge and intention, cognitive fit theory, media richness, and sticky information. Our work is combining different aspects of these theories to answer the question: “How innovative knowledge of customers influences their recommendation intentions, when engaged in innovative activities?”

### Customer Knowledge

Customer knowledge is an important part of firm knowledge. Nonaka et al. (2000) classified customer knowledge into “explicit knowledge,” expressed in formal and systematic language, and “tacit knowledge,” highly personal and difficult to formalize. Customer knowledge can be also classified into the knowledge about customers—related to potential customers or customer segments, as well as knowledge about individual customers and the knowledge possessed by customers—about product ranges and the wider context and marketplace into which products and services are delivered (Rowley, 2002). Smith and McKeen (2005) proposed the third dimension, called the *knowledge cocreation with customers*, which involves asking customers to collaborate and interact closely in knowledge cocreation. Of the three dimensions of customer knowledge management, knowledge cocreation with customer turns out to be the strongest predictor of innovation quality and speed (Taghizadeh et al., 2017). Brucks (1985) believes that customer knowledge should be examined based on “subjective,” knowledge refers to the confidence consumers have about the products, and “objective,” knowledge refers to their actual knowledge of the product. So, customer knowledge covers a wide, diverse, and complex field, and a generally agreed definition has not been yet formed. Thus, any discussion on customer knowledge needs to be placed in a specific context. For example, Fidel et al. (2015) argue that “customer knowledge management” may be affected by innovation orientation and customer collaboration, which could further affect marketing results. They sustain that innovation orientation has a direct and positive impact on customer knowledge management.

### Relationship Between Knowledge and WOM

The literature has reported inconsistent findings regarding the impact of customer knowledge on WOM recommendations. For example, Brucks (1985) found a negative correlation between the effort placed on information collection and the actual knowledge of the information seekers; his approach was applied by many scholars to study the WOM recommendations. Conversely, Gilly et al. (1998) empirically found neither a positive nor a linear relationship between product knowledge and WOM recommendations. Considering non-interpersonal factors, Bansal and Voyer (2000) found that receiver’s expert and sender’s expertise influence WOM on service purchase decisions. Chiou et al. (2002) found the effect of customer knowledge on loyalty may be mediated by the level of satisfaction. Lee and Koo (2012) argue that the effect of WOM attributes and valence would be naturally moderated by the subjective knowledge of their customers. That is to say, the literature on customer knowledge and WOM recommendations offers mixed results.

Recently, more literatures considered have examined the impact of customer knowledge management on performance (Fidel et al., 2015) and demonstrated that customer knowledge management improved firm product innovation performance (Falasca et al., 2017; Guan et al., 2018) and had been considered for the new product development and innovation quality (Chang and Taylor, 2016; Taghizadeh et al., 2017).

These studies above, however, did not examine how the customer knowledge affects consumer recommendation when customers engaged in innovative activities. This study extends prior research by examining the relationship according to cognitive fit theory.

### Cognitive Fit Theory

Cognitive fit theory suggests that information processing by individuals is more efficient and effective if they can employ appropriate cognitive processes based on the information provided (Teets et al., 2010; Chan et al., 2012). We also can employ cognitive fit theory in the customer innovation processes, and the key of the process is customer knowledge. As an example of such processes, Fisher and Amabile (2009) discuss the creative model process, which is the improvisational creativity in a business organization for gaining progress in its area of operation. They point out that, to facilitate this type of creativity, a person must acquire the necessary expertise. We study the moderating effect of GMs and materials, which are key factors that determine customers’ level of engagement in innovative activities based on the cognitive fit theory.

### The Moderating Effect of Guidance Methods

Besides the cognitive fit theory, we use media richness theory, which maintains that performance can be improved when people use “richer” media for their tasks, to explain the moderating effect of GMs. Guidance methods refer to a firm’s use of multiple cues, such as words, pictures, and video, to help customers experience the innovative products or service. Guidance methods reflect the multiple cues, which refer to the ability to transmit multidimensional information into a range of meanings according to measure media richness theory (Daft and Lengel, 1984). Customers with different levels of knowledge (cognitive variations) will most likely respond to media richness differently. Namely, both customer knowledge and GMs may jointly affect customers’ recommendation intentions.

### The Moderating Effect of Materials Provided

We also use sticky information theory, which maintains that the transmission of information between subjects is slow and costly (Sánchez-González et al., 2009), to explain the moderating effect of information-transmitting materials. According to this theory, it may therefore be prudent for businesses to provide an innovation toolkit that allows customers to select designs of their preferences. Businesses often provide design materials to guide their innovation activities (e.g., EQXIU product application design, the MI online TV experience, and the Lenovo ThinkPad S design). From the firm’s perspective, a key factor influencing recommendation intentions is whether the materials provided to customers match their knowledge levels. The materials offered for innovation activities vary extensively. Some offer only limited choices in order not to overstrain customers (Huffman and Kahn, 1998), whereas others offer a virtually infinite solution space in order to enable closer preference fit (von Hippel and Katz, 2002).

In summary, despite much research on customer knowledge and WOM, there are shortcomings as follows: innovative customers’ recommendation has not received enough attention, and the perspective of firm design has not been taken into consideration. Compared with other customer perspectives, firm design perspective could help firms to do better customer knowledge management in innovative platforms. In other words, the factors of firm design are more likely to play an important role in the relationship between customer knowledge and their recommendations. Moreover, the experimental research has not been used in these fields, and the theory tended to be single, and we try to draw on experimental research and combine different aspects of cognitive fit theory, media richness, and sticky information to answer the question of what is the impact of customer knowledge over their recommendation intentions from firm design perspective. Table 1 is positioning our research into the relevant literatures.

**TABLE 1:** Proposed design positioned into the literature.

**Authors**	**Focus**	**Solution methodology**	**Objective**	**Perspective**
Park and Kim, 2008	Consumer expertise, cognitive type	Analysis of variance	↑ Online consumer review	Customer review
Kumar et al., 2010	Consumer engagement, lifetime, knowledge	Theoretical research	↑ Customer value	Customer value
Lee and Koo, 2012	Review credibility, adoption, subjective knowledge	Analysis of variance	↑ WOM	Customer review
Fidel et al., 2015	Innovation orientation, customer knowledge management	Structural equation model	↑ Marketing results	Customer engagement
Chang and Taylor, 2016	Customer participation, new product innovativeness	Meta-analytic path analysis	↑ New product development	Customer engagement
Falasca et al., 2017	Customer knowledge management	Structural equation model	↑ Firm product innovation performance	Customer engagement
Taghizadeh et al., 2017	Customer knowledge, customer engagement	Structural equation model	↑ Innovation quality	Customer engagement
Guan et al., 2018	Customer expertise	Analysis of variance	↑ Customer knowledge sharing	Customer engagement
This research	Customer knowledge, guidance methods, materials provided	Experiments research	↑ Customers’ recommendations	Firm design

## Proposed Research Framework

Following the insights given by the customer knowledge literature, in the proposed research framework, we divided the innovation-related knowledge of customers into subjective and objective knowledge and discuss them under specific experimental design and scenarios. We define *subjective knowledge* as customer’s perception of their understanding of innovative products and involve a process of self-assessment, whereas *objective knowledge* is the knowledge that customers possess about the innovative activities.

Researchers have primarily focused on the relationship between customer knowledge and WOM recommendations from the customer’s perspective. In this article, we study the impact of customer knowledge and WOM recommendations from the firm’s perspective. We argue that the relationship between customer knowledge and WOM recommendations may be affected by the design of firm activities. When invited to participate in innovative activities, customers with high levels of knowledge will achieve a higher level of satisfaction with their products, which will directly affect their willingness to make WOM recommendations. This leads us to the first hypothesis of the study:

**Hypothesis 1**: *The level of customer knowledge will be positively correlated with their recommendation intentions*.

Drawing from the cognitive fit theory studies, we conjecture that when innovative design matches the level of customer knowledge, the customer’s effort will be lower, thereby resulting in a more confident customer who would be more willing to make recommendations. Conversely, if the innovative activity does not match the customer’s knowledge level, customer’s innovation costs will be high, leading to less willingness to make recommendations.

As adequate knowledge and experience are needed to understand more complex literature GMs, expert customers, who are more likely to have the knowledge to handle such materials, may be more drawn toward GM literature. The successful handling of such literature may in return reinforce and boost the confidence levels of the expert customers. Therefore, literature on GMs is better suited for expert customers. In contrast, novice customers who are less capable of obtaining and using external support tend to prefer straightforward graphical GMs to reduce their innovation cost while raising their creative confidence. In short, graphical GMs provide novices with an intuitive operational model better suited to their knowledge levels. Thus, GMs may be an important moderator variable, which lead to the following hypotheses:

**Hypothesis 2**: *The GMs will have moderating effects on the relationship between customer knowledge and recommendation intentions*.
**Hypothesis 2a**: *Novice customers will exhibit higher recommendation intentions when they are provided with graphical guidance*.
**Hypothesis 2b**: *Expert customers will exhibit higher recommendation intentions when they are provided with literature guidance*.

In our study, we divided the materials into *template* and *no-template* materials. Given that customers with different knowledge levels will seek different types of information (Park and Kim, 2008), those with higher levels of knowledge are likely to assess the information based on their proficiency, and their information processing is likely to differ from those of low levels. Through template and no*-*template materials, firms can successfully reduce information stickiness. However, when adopting such an approach, management needs to recognize that customer knowledge and the materials provided may have an interactive effect on customers’ recommendation intentions.

Customers with better awareness and application of the products, “the experts,” tend to prefer no*-*template materials, as opposed to template materials, as they can make full use of their personal knowledge, embody the value of the knowledge held, and trigger recommendations based upon such self-recognition. Conversely, customers with lower levels of product knowledge, “the novices,” are unfamiliar with the products, such that they will have less ability to use and process the materials and will be more inclined to make reactionary decisions (Alba and Hutchinson, 1987). Thus, compared with no-template materials, template materials may be better suited to novice customers. Template materials can, to a certain degree, mitigate customers to suspect their knowledge level and make customers more confident to recommend.

From above, we expect the materials used to transmit information to have a moderating effect, which leads to the following hypotheses:

**Hypothesis 3**: *The types of materials provided will have moderating effects on the relationship between customer knowledge and recommendation intentions.*
**Hypothesis 3a**: *Novice customers will exhibit higher recommendation intentions when they are provided with template materials*.
**Hypothesis 3b**: *Expert customers will exhibit higher recommendation intentions when they are provided with no-template materials*.

**FIGURE 1:**
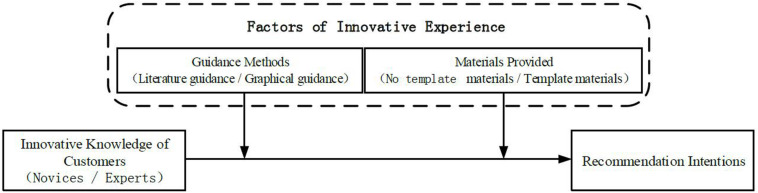
Research Framework and hypothesized relationships.

The research framework of our study is presented in Figure 1.

## Experimental Design and Data Analysis

To test the hypothesis formulated in *Proposed Research Framework*, we designed a two-step experiment:

**Step 1:** A preliminary experiment was designed to identify the appropriate online platform to be used.
**Step 2:** Two experimental studies have been designed, using the best platform identified in Step 1, to test the relationship between (1) GM and customer knowledge and (2) type of material used and customer knowledge, controlling for GM.

### Preliminary Experiment

The preliminary experiment of this study was undertaken in the behavioral laboratory of the authors’ university and involved 40 graduate students (55% females) with average age of 24.7 years. We presented a set of innovation platforms (web sites that allow customers to participate in the production of personalized products) and asked the participants to rate their level of interest and recommendation intentions based on a 5-point Likert scale. From the results summarized in Table 2, we determined the most appropriate innovation platform to be used in the next steps of the study.

**TABLE 2:** Innovation platforms, level of interest, and recommendation intentions.

**Variables**	**Kagirl**	**Vxiu**	**EQXIU**	**RabbitPre**	**Chuye**
**Level of interest**					
Mean	3.60	4.00	***4.20***	3.90	3.70
SD	1.66	1.60	1.32	1.35	1.40
**Recommendation intentions**					
Mean	3.50	3.90	***3.90***	3.70	3.40
SD	1.63	1.73	1.58	1.27	1.59

In preliminary experiment, we presented a set of innovation platforms (web sites that allow customers to participate in the production of personalized products), and asked the participants to rate their level of interest and recommendation intentions based on a 5-point Likert scale. From the results summarized in Table 2, we determined the most appropriate innovation platform to be used in the next steps of the study. And the results show that the EQXIU platform achieved the highest score for both variables of interest, with mean interest of 4.20 and mean recommendation intentions of 3.90.

To measure the objective knowledge of participants in using the EQXIU platform, we uncovered participants’ knowledge levels through in-depth interviews, which involved questions such as “What knowledge has helped you complete today’s experiment?” and “Which aspects of knowledge should you master in order to complete today’s innovation work?” Finally, the participants were asked to rate the most important elements of the design work on a 5-point Likert scale. Table 3 shows the key factors, and the top five are: (1) typesetting and editing (with mean of 4.6); (2) slide production (with mean of 4.5); (3) applications and functions of EQXIU platform (with mean of 4.3); (4) animation production (with mean of 4.3); and (5) aesthetic design (with mean of 4.2).

**TABLE 3:** The objective knowledge of customers based on EQXIU innovation platform.

**Description**	**Mean**	**SD**
Applications and functions of the platform	***4.3***	1.62
Similar platform production experience	3.6	1.54
Computer operation	4	1.55
Typesetting and editing	***4.6***	1.76
Language skills	3.5	1.24
Color match	3.8	1.66
Aesthetic design	***4.2***	1.35
Slide production	***4.5***	1.54
Information collection	3.7	1.44
Animation production	***4.3***	1.56
Material processing	4.1	1.43
Innovation experience	3.4	1.34

We follow the approaches of Brucks (1985) and Pieniak et al. (2010) to measure the objective and subjective knowledge, using both the collected data and in-depth interviews. Table 4 shows the indicators used to measure these types of knowledge. In order to exclude any non-qualifying questions, the knowledge measurement was also tested by a reverse option, which was negatively worded.

**TABLE 4:** Questions to assess customer’s knowledge and recommendation intentions.

**Sample responses**	**Sources**
Subjective knowledge	Park and Kim, 2008
I will actively seek to obtain product knowledge of EQXIU.	
If someone were to ask me, I would be able to provide advice on EQXIU product manufacturing.	
I can identify the product applications and functions of EQXIU.	
Compared with other people, I believe I have richer design experience of EQXIU products.	
Compared with other people, I believe I have richer experience of EQXIU product publicity.	
I don’t have a very good understanding of EQXIU products.	
Objective knowledge	Park and Kim, 2008
I am familiar with the applications and functions of the EQXIU platform.	
I have mastered the knowledge of layout editing.	
I have mastered the knowledge of aesthetic design.	
I have mastered the knowledge of slide production.	
I have mastered the knowledge of animation production.	
Recommendation intentions	Carroll and Ahuvia,
I would be willing to show my product to others.	2006; Keng et al., 2007
I would be willing to discuss my product with others.	
I would be willing to recommend my product to friends and relatives.	
I will introduce EQXIU and its related products to friends and relatives.	
I will recommend the products of EQXIU to friends and relatives.	

*The questionnaire used a 5-point Likert scale, where “1” indicates “strongly agree” and “5” indicates “strongly disagree.”*

### First Experimental Design

The primary aims of the first experiment developed were to investigate the impact of customer knowledge on recommendation intentions using the EQXIU platform and to examine the moderating effect of the GMs provided by the business on the relationship between customer knowledge and recommendation intentions. These two goals are directly associated with the first two hypotheses in our study.

#### Experimental Procedure

The first experiment used a mixed design based on the two customer knowledge levels (novices and experts) and the two types of GMs (literature and graphical). A sample of 88 undergraduate students (as opposed to 40 graduate students in preliminary experiment) participated in the study, and they were randomly divided into four groups. Following Park and Kim (2008) approach, we divide the participants into two groups (novices and experts) based on their scores, with respect to the EQXIU products. This partition method is commonly used in the field of consumer knowledge (Alba and Hutchinson, 1987; Park and Kim, 2008). Meanwhile, the guidance materials offered on the EQXIU platform were tailored for the two types of GMs. The guidance via literature provided the detailed steps only in text format (Supplementary Appendix 1), whereas the guidance via graphics provided the same text with a graphical illustration of the operations (Supplementary Appendix 2).

The participation in the study was voluntary, and the participants were not required to be familiar with the EQXIU platform before signing up for the study. As part of the study, participants were first asked to read the introduction to EQXIU on the official website and also refer to the Baidu Encyclopedia (Online Interactive Encyclopedia^1^). This was followed by the participants’ self-assessment of their level of knowledge of the EQXIU product through the completion of a questionnaire. The participants were then asked to register for a personal account at www.eqxiu.com, and create an H5 micro-scene product on the theme of “publicity for Huawei.” According to their combined average score on their subjective and objective knowledge of the EQXIU product, the participants were divided into “novices” and “experts,” with each group containing 44 participants. Further, the participants from each group were randomly assigned to one of the two GMs: literature or graphical.

The participants assigned to the literature guidance group read the text provided prior to engaging in the production of the “Huawei publicity” topic. Similarly, the participants assigned to the graphical guidance group were provided with both the text and the associated graphical illustrations. The successful completion of the assignment required participants to have produced a personal H5 mobile micro-scene on the EQXIU website, with the work fully completed and submitted within the stipulated time. At the end, participants completed a questionnaire on their recommendation intentions, which measure referred to Carroll and Ahuvia (2006) and Keng et al. (2007).

#### Manipulation Check

We used a 2 × 2 analysis of variance (ANOVA) on subjective and objective knowledge within the novice and expert groups; the analysis results revealed significant differences between the subjective knowledge score (*M*_expert_ = 3.84 > *M*_novice_ = 2.74, *p* < 0.01) and the objective knowledge score (*M*_expert_ = 3.76 > *M*_novice_ = 2.92, *p* < 0.01). In order to carry out a manipulation check of the effectiveness of the GMs on customer knowledge, the participants were asked to rate a single question (“Depending on your level of knowledge, how difficult is the guidance for you?”) on a 5-point Likert scale, with 1 denoting “very difficult” and 5 signaling “very easy.” The results obtained from the novice group showed that the average score for the graphical GM is higher than the average score for the literature GM (*M*_graphical guidance_ = 3.41 > *M*_literature guidance_ = 2.14, *p* < 0.01), whereas those obtained from the expert group indicated a reversed effect, with an average score higher for the literature GM (*M*_literature guidance_ = 4.32 > *M*_graphical guidance_ = 3.23, *p* < 0.01). Thus, literature guidance was easier for customers with higher knowledge levels (the experts), and graphical guidance was more suitable for customers with lower knowledge levels (the novices).

We were also interested in determining whether the literature or graphical GM provided a better match with the knowledge level of the expert or novice group. We used an independent-sample *t* test to verify whether there were any significant differences in the scores between the two GMs, and also a paired-sample *t* test to verify whether the two GMs showed any significant differences when paired with the two different knowledge levels (Table 5).

**TABLE 5:** Guidance method manipulation check.

**Guidance methods**	**Novice group**	**Expert group**	***t* value^a,c^**
Graphical guidance	3.41	4.32	–4.60***
Literature guidance	2.14	3.23	–4.91***
*t* value^b,c^	6.38***	5.26***	–

^*a*^*t* value refers to the independent-sample *t* test. ^*b*^*t* value refers to the paired-sample *t* test. ^c^***p < 0.001.

The results synthetized in Table 5 show that the novice group ratings for the graphical GM are significantly higher than the ones for the literature GM (*t* = 6.38, *p* < 0.001), but they are lower than the ratings given by the expert group (*t* = -4.60, *p* < 0.001). On the other hand, experts’ rating on literature guidance is lower than on graphical guidance (*t* = 5.26, *p* < 0.001), but higher than the ones of the novice group (*t* = -4.91, *p* < 0.001). In conclusion, the graphical GMs are more appropriate for the novice customers, given their current level of knowledge. Even though expert customers also like the graphical guidance, they have a higher degree of appreciation for the literature guidance relative to novices.

#### Results

Before testing our hypotheses, we first examined the validity and reliability of the scales used to collect the data. We found the Cronbach α associated with the customer knowledge scale to be 0.86, and all of the factor loadings associated with the confirmatory factor analysis were between 0.80 and 0.85. The recommendation intentions scale had a Cronbach α of 0.91, and all factor loadings were between 0.80 and 0.92. Thus, the scales used in this study are reliable and valid.

We performed statistical analyses to assess the severity of common method bias. First, a Harmon one-factor test (Podsakoff and Organ, 1986) was conducted on the crucial variables in theoretical model including objective knowledge, subjective knowledge, and recommendation intentions. Results from this test showed that three factors were present, and the most covariance explained by one factor was 39.37%, indicating that common method bias is not a likely contaminant of our results. Second, we followed the method proposed by Malhotra and Patil (2006) to conduct a confirmatory factor analysis loading all items on a single factor and examined the fit indices. The χ^2^ difference between the single-factor model and our measurement model was statistically significant (*Δχ*^2^ = 354.9, Δ*df* = 3, *p* < 0.00), thereby indicating that common method bias was not a potential threat (Supplementary Appendix 4).

**FIGURE 2:**
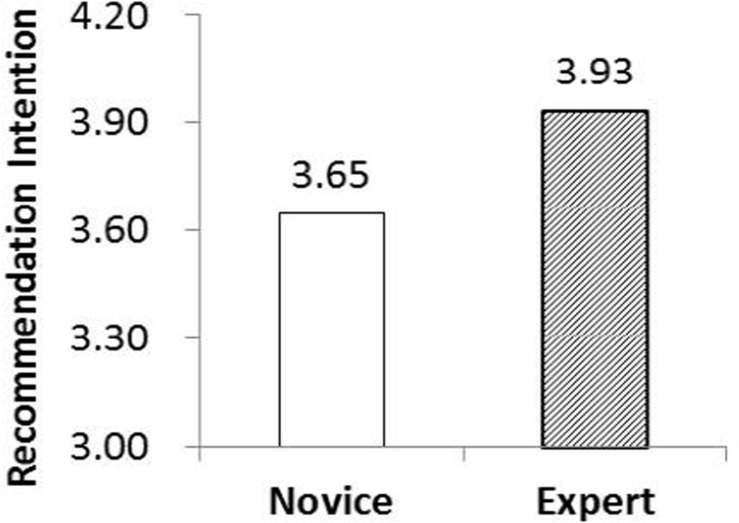
Main effect of customer knowledge on WOM intentions.

The average score for the knowledge level of the novice group in the first experiment was 2.83, whereas that of the expert group was 3.80. The main effect of customer knowledge on recommendation intentions was significantly positive (*p* < 0.05). Specifically, as shown in Figure 2, the recommendation intentions score was higher in the expert group (*M* = 3.93) than in the novice group (*M* = 3.65). These results support Hypothesis 1, that the level of customer knowledge is positively correlated with their recommendations intentions.

Table 6 shows that the recommendation intentions of expert customers are higher than those of the novice customers, but the differences between graphical guidance and literature GMs are not significant (*p* > 0.05). The interaction effect between customer knowledge and GMs is not statistically significant either (*p* > 0.05). We conducted moderated multiple regression analyses using Process Macro (Model 1) with 5,000 bootstrap resamples (Hayes, 2013) to further test the interaction effect between customer knowledge and GMs. Overall, regressing customer knowledge, GMs, and their interaction term indicated that the interaction effect on recommendation intentions was not significant [*b* = -0.39, SE = 0.22, 95% confidence interval (CI) = -0.83 to 0.04] (Supplementary Appendix 5).

**TABLE 6:** Customer knowledge, guidance methods, and their interactions.

**Independent variables**	**SST**	***df***	**MS**	***F***	***P* value**
Customer knowledge	1.79	1	1.79	6.93	0.01
Guidance methods	0.61	1	0.61	2.36	0.12
Customer knowledge × guidance methods	0.85	1	0.85	3.30	0.07

**FIGURE 3:**
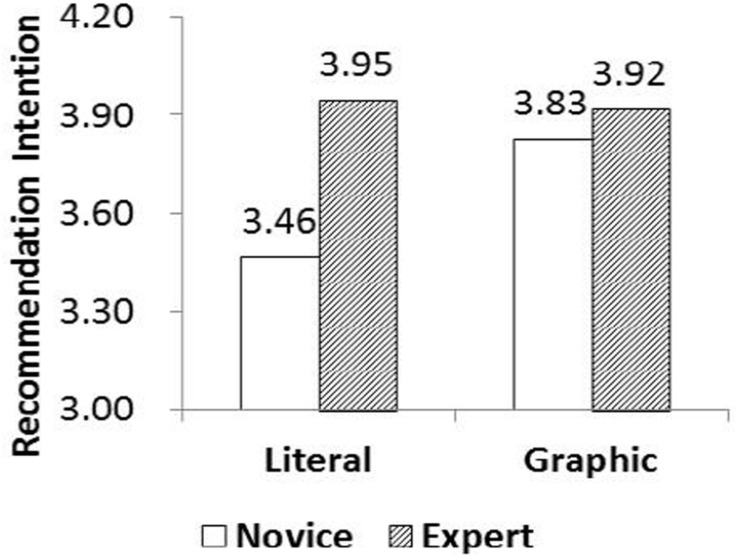
Interaction between customer knowledge and guidance methods.

In Figure 3, we can see that the recommendation intentions of novices increase when the graphical GM is used (*M*_graphical_ = 3.83, SD = 0.62; *M*_literature_ = 3.46, SD = 0.53; *p* < 0.05). The results support Hypothesis 2a, that the novice customers exhibit higher recommendation intentions when they are provided with graphical guidance.

However, for expert customers, no recommendation intention differences are found between the two GMs (*M*_graphical_ = 3.92, *M*_literature_ = 3.95, *p* > 0.05). Hypothesis 2b is not supported by our data, so we cannot conclude that *expert customers will exhibit higher recommendation intentions when they are provided with literature guidance*.

In conclusion, the recommendation intentions of novice customers with graphical guidance were higher than with literature guidance. However, those of expert level were the same regardless of the GM used. Thus, Hypothesis 2 is only partially supported by our findings.

### Second Experimental Design

To avoid possible influences of creative themes on our experimental results, we used the second experiment to retest Hypothesis 1 under a new creative theme. The data collected were also used to test Hypothesis 3 and assess the moderating effects of different materials (template or no template) on the relationship between customer knowledge and their recommendation intentions. To ensure that our experimental results are not influenced by the GMs, neither of the two methods was included in this experiment.

#### Experimental Procedure

The second experiment was designed by taking into consideration the customer knowledge levels (novice and expert) and the type of materials provided (template and no template). The data were collected from 96 undergraduate students, and the participants were randomly divided into four groups. The independent variables considered in the analysis were customer knowledge and materials provided, whereas the dependent variable was recommendation intentions. The stimuli provided in this experimental were template and no-template materials (Supplementary Appendix 3). Expert customers tend to choose no*-*template materials to reflect their personality/characteristics and to enable them to create freely, whereas template materials are often preferred by customers who rely upon outside help for their creativity (i.e., use of existing creation-inspiring materials). The no*-*template materials provided in the second experiment allowed participants to use their own photographs and music, whereas the template materials allowed only the use of creative theme-related materials provided by the authors.

Similar to the first experiment, the participants were first asked to read the introduction from the EQXIU website and Baidu.com. This was followed by the participants’ self-assessment of their level of knowledge with the EQXIU product through the completion of a questionnaire. Next, the participants registered for a personal account at www.eqxiu.com and created an H5 micro-scene product on the theme of “Teacher’s Day, let us express our appreciations.”

According to their combined subjective and objective knowledge score of EQXIU, the participants were again divided into novices (sample with 44 participants) and experts (sample with 52 participants). The participants in each group were then randomly assigned to no*-*template materials or template materials. The participants assigned to receive no*-*template materials used their own photographs and music for their innovative activity, whereas the participants assigned to receive templates used the theme-related materials provided by the authors (Supplementary Appendix 3). Upon completion of their tasks, the participants were asked to complete a questionnaire on their recommendation intentions.

#### Manipulation Check

Similar with the first experiment, we used a 2 × 2 ANOVA to analyze the subjective and objective knowledge of the novice and expert groups. The results obtained indicate that there are significant differences between experts and novices, in both subjective knowledge score (*M*_expert_ = 3.89 > *M*_novice_ = 2.63, *p* < 0.01) and objective knowledge score (*M*_expert_ = 3.85 > *M*_novice_ = 2.81, *p* < 0.01).

In order to carry out a manipulation check of the effectiveness of the materials provided on customer knowledge, the participants were asked to rate a single question (“Depending on your level of knowledge, how much does the provided material help you complete the experiment?”) on a 5-point Likert scale, with 1 representing “very little help” (difficult task) and 5 representing “much help” (i.e., very easy task). The analysis revealed that, for the novices group, the average score was higher for the template materials (*M*_template_ = 4.14 > *M*_no–template_ = 2.55; *p* < 0.01), whereas for the experts group, the average score was higher for the no*-*template materials (*M*_no–template_ = 4.08 > *M*_template_ = 2.54; *p* < 0.01). So, we can conclude that novices prefer template materials, whereas experts prefer no*-*template materials.

To further examine if the template or no*-*template materials provide a better match with the knowledge level of the experts or novices, we used an independent-sample *t* test and a paired-sample *t* test to carry out the analysis. The results summarized in Table 7 confirm that novices prefer template to no*-*template materials, and the usage level of the template materials is higher for this group when compared with the experts. Alternatively, experts use more no-template materials than templates, and their usage level is higher than that of novices. This indicates that the materials provided need match the knowledge levels of the customers: novices like template materials, whereas experts favor no*-*template materials.

**TABLE 7:** Manipulation check on materials provided.

**Materials provided**	**Novice group**	**Expert group**	***t* value^a,c^**
Template materials	4.14	2.53	7.19***
No template materials	2.55	4.08	–7.76***
*t* value^b^	8.73***	–7.35***	–

^*a*^*t* value refers to the independent sample *t* test. ^*b*^*t* value refers to the paired-sample *t* test. ^c^***p < 0.001.

#### Results

We performed statistical analyses to assess the severity of common method bias. A Harmon one-factor test (Podsakoff and Organ, 1986) was conducted on the crucial variables in theoretical model including objective knowledge, subjective knowledge, and recommendation intentions. Results showed that three factors were present, and the most covariance explained by one factor was 44.42%, indicating that common method bias was not a likely contaminant of our results. In addition, the χ^2^ difference between the single-factor model (Malhotra and Patil, 2006) and our measurement model was statistically significant (*Δχ*^2^ = 317.7, Δ*df* = 3, *p* < 0.00), providing further evidence that common method bias did not influence the significance of the results (Supplementary Appendix 4).

**FIGURE 4:**
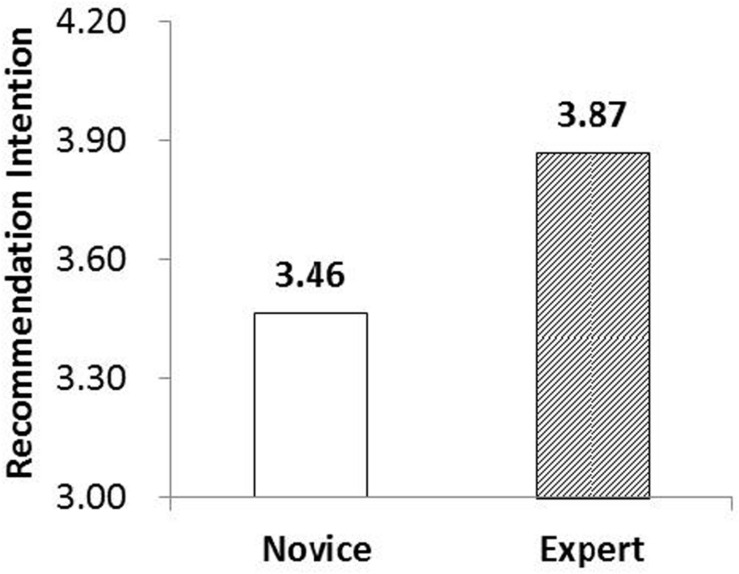
Main effect of customer knowledge on WOM intentions.

Our analysis revealed that the main effect of customer knowledge on recommendation intentions in the second experiment is significantly positive (*p* < 0.01). Specifically, the recommendation intentions score was found to be higher for the experts group (*M* = 3.87) when compared with that of the novices group (*M* = 3.46), as shown in Figure 4. These results provide additional evidence to support Hypothesis 1. Table 8 shows that the moderating effect of the materials provided between customer knowledge and recommendation intention is also statistically significant (*p* < 0.01). We conducted moderated multiple regression analyses using Process Macro (Model 1) with 5,000 bootstrap resamples (Hayes, 2013) to further test the interaction effect between customer knowledge and materials provided. Overall, regressing customer knowledge, materials provided, and their interaction term indicated a significant interaction effect on recommendation intention (*b* = -0.73, SE = 0.20, 95% CI = [-1.14 to -0.33]) (Supplementary Appendix 5).

**TABLE 8:** Customer knowledge, materials provided, and their interactions.

**Independent variable**	**SST**	***df***	**MS**	***F***	***P* value**
Customer knowledge	4.91	1	4.91	22.79	0.00
Materials provided	0.16	1	0.16	0.74	0.38
Customer knowledge × materials provided	2.76	1	2.76	12.84	0.00

**FIGURE 5:**
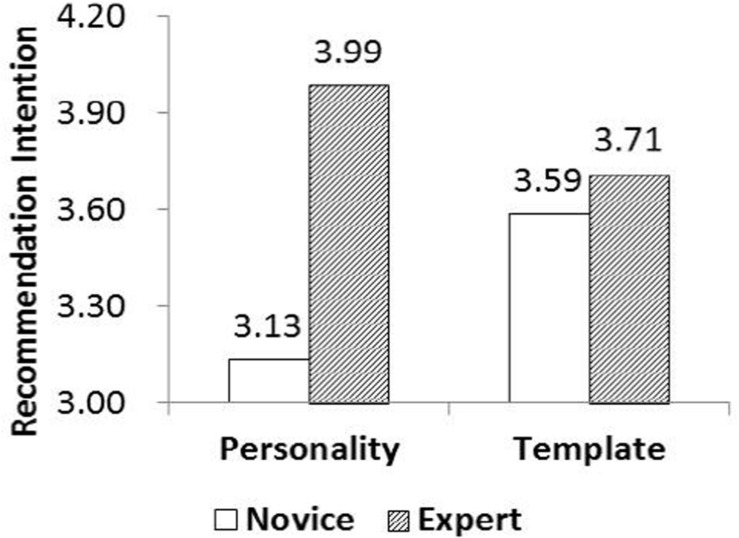
Interaction between customer knowledge and materials provided.

Figure 5 shows that novices prefer the template materials (*M*_no–template_ = 3.13 < *M*_template_ = 3.59, *p* < 0.01), whereas the experts prefer the no*-*template materials (*M*_no–template_ = 3.99 > *M*_template_ = 3.71, *p* < 0.05). Thus, the recommendation intentions of novices given templates were higher than those given no*-*template materials, whereas the recommendation intentions of experts given no*-*template materials were higher. In conclusion, Hypotheses 3, 3a, and 3b are supported, suggesting that the *types of materials provided have a moderating effect on the relationship between customer knowledge and recommendation intentions*. Novice customers will exhibit higher recommendation intentions when provided with template materials, whereas expert customers will exhibit a higher intention when provided with no*-*template materials.

## Discussion and Implications

### Discussions

On both experiments, our results indicate that knowledge of innovative products/services among customers has significant effects on their recommendation intentions: the higher their knowledge levels, the higher their recommendation intentions. The innovative products made by the “customers” in our experiment were immediately visible and available, thus consistent with the results from Chiou et al. (2002) that customers who have higher knowledge levels would increase their recommendation intentions. We also conjecture that, when innovative design matches the level of customer knowledge, the customer’s effort will be lower, thereby resulting in a more confident customer who would be more willing to make recommendations. Conversely, if the innovative activity does not match customer’s knowledge level, customer’s innovation costs will be high, leading to less willingness to make recommendations.

However, the results of the interaction effect between customer knowledge and GMs were not statistically significant. If firms could provide detailed guidance to facilitate customers experiencing the innovation process, it may enhance customers’ confidence in completing the task. The guidance could apprise customers about the innovation task and save learning time and effort. But the guidance provided (literature or graphical guidance) had no significant impact on the recommendation intentions, although expert customers’ recommendation intentions were consistently higher than those of novices. These findings are opposite to those reported on general customers by Park and Kim (2008). One possible explanation for this could be the dependence of such activities on customers’ prior knowledge and experience, given the innovative experience is carried out under a network platform using a toolkit. Under such circumstances, firm’s guidance may have only little effect, whereas customers depend more on their own knowledge to tackle innovative design. On the other hand, the content of guidance provided may motivate customers to participate in the innovative experience with the EQXIU platform, but did not focus on the operational or technical details of the task. Thus, during the task engagement, customers relied more on their prior knowledge or sought help using the internet, rather than choosing to view the specific guidance provided. This implies that experts have sufficient confidence in their ability to understand both the simple and complex GMs. For novices, graphical guidance does reduce their innovation costs, but they have insufficient confidence to use their knowledge/experience to engage in innovation. Thus, the interaction effect between customer knowledge and methods of guidance is found to be insignificant.

The second experiment has demonstrated a significant interaction, which is found between customer knowledge and the materials provided. Specifically, the recommendation intentions among novices provided with template materials are higher than those with no*-*template materials. Conversely, the recommendation intentions of experts provided with no*-*template material are higher than those with template materials. The results again confirm the findings of Park and Kim (2008) that different levels of knowledge among customers led to different quest of information. Template materials are a better fit to novices, a finding resonating with customer awareness discussed in Teets et al. (2010). Low-knowledge novices are more relaxed and confident with the template design process, thereby reducing their doubts on their personal knowledge and encouraging them to make recommendations. No*-*template materials are better suited to customers with higher levels of innovative knowledge; such customers can make full use of their knowledge and reflect the value of such knowledge, which can ultimately trigger WOM recommendations.

### Implications

First, considering the influence of product life cycles, customers who are early adopters and who actively participate in product innovation will invariably have higher knowledge levels, whereas customers involved in the mainstream market (i.e., maturity stage of the product life cycle) are likely to have lower knowledge levels. From the perspective of customers’ participation, those regularly participating in innovative experience will invariably have higher knowledge levels. For example, users who frequently post or respond within the innovative community often are expert customers. Conversely, customers with low participation levels often have low knowledge levels. From the platform perspective, customers who can expertly operate and efficiently use tools for innovative activities in the platforms often have higher knowledge.

Second, businesses can increase customers’ knowledge levels by improving the design of their innovative activities. We found that the recommendation intentions of experts were higher than those of novices, regardless of whether they were provided with literature or graphical GMs. Although the interaction between the GMs and customer knowledge is not significant, the influence of innovative customer knowledge levels on their recommendation intentions is nevertheless verified once again.

Business could also provide appropriate levels of support for their customers, such as improving the toolkits for innovation, optimizing the innovative design interface, offering customers tips and guidance throughout the design process, and providing them with advance training. Not only will such approach help customers to readily understand and handle the related innovative tasks, but also the increased support provided to customers during such tasks will help improve their knowledge levels, which may generate spontaneous WOM recommendations.

Third, GMs should be established according to customer knowledge levels. Knowing that no*-*template materials can lead to customers creating products that are specifically tailored to their personal preferences, firms could provide expert customers (whom they identify as possessing higher knowledge levels) with greater design freedom, thereby potentially generating WOM recommendations. For example, many firms in the computer games business provide users with complete freedom to design their own toolkits, an approach that can have significantly positive impacts on the developers (Prügl and Schreier, 2006). However, we have shown that template materials are better suited to customers with low knowledge levels. Thus, firms should predefine specific functions in the toolkits they provide to ensure that the templates match customer preferences, potentially through a monitoring system that captures customer information on knowledge levels and makes matching recommendations.

## Conclusion and Limitations

By combining cognitive fit, media richness, and sticky information theories, we conducted two experiments to examine the influence of customers’ innovative knowledge levels on their experience during innovative activities. Our results showed the existence of significant differences between the novice customers and expert customers in their innovative experiences. Understanding how customer knowledge and external factors (e.g., GMs) connect will help identify the essential factors that determine each type of customers to help with WOM referrals.

We also found that the interaction between customer knowledge and the GMs provided is not significant. However, novices feel more comfortable when provided with template materials, whereas experts prefer the experiences guided by no*-*template materials. Businesses should take note of the impact of innovative customer knowledge on their experiences and preferences whenever designing innovative activities. Firms could improve the design of their innovative experience by enhancing customer knowledge levels. Finally, firms should design suitable material modes (template or no template) to match customer knowledge levels in order to maximize customer experiences of innovation platforms.

We would also like to acknowledge some limitations of the current study. One limitation of our study is the relatively small sample size for the two-step experimental analysis—only 40 students were used to identify the appropriate online platform, and 88 and 96 students were recruited in the two experiments run to test our hypothesis. Even though the three sample sizes can be considered small, the results of our findings can be generalizable given that the characteristics of the participants mimic the ones at the population level. Future work targets the extension of our research to people from different groups and professions. Second, we select the platform basing on the customer’s interest and recommendation intentions. However, the knowledge of the platform, technical background, and many other factors will have a significant impact on the selection of the final platform. Future research can consider these factors and control the knowledge and technical background. The third, the selection of the types of GM, which is considered textual and graphical, is too single; future research could compare textual and multimedia-oriented material, such as video, Flash, and so on, instead of them, and include the age of the subjects as a control variable. Finally, using the example of EQXIU in this study, it has proven difficult to completely reflect the actual innovation situation and market environment, but it offers a good example of innovative experience. Future research could engage in repeated experiments or controls using various instruments to examine the impact of innovative customer knowledge on their recommendation intentions, across different innovation platforms.

## Data Availability Statement

All datasets generated for this study are included in the article/Supplementary Material.

## Ethics Statement

This study was reviewed and approved by the Internal Review Board of School of Management, Guangdong University of Technology. Written, informed consent was obtained from participants. The participants were notified that all of their responses would only be accessible to the researchers.

## Author Contributions

FZ and DZ designed experiments. FZ and ML carried out experiments. FZ, DZ, and ML analyzed experimental results and wrote the manuscript.

## Conflict of Interest

The authors declare that the research was conducted in the absence of any commercial or financial relationships that could be construed as a potential conflict of interest.

## Footnotes

1 www.baike.baidu.com
