# Supplementary material for: The Impact of the Innovative Knowledge of Customers on Their Recommendation Intentions
Source: Front Psychol. 2020 Jun 3;11:979. doi: 10.3389/fpsyg.2020.00979 (PMC7283618; doi:10.3389/fpsyg.2020.00979)
Supplement: Supplementary file 1 [file Data_Sheet_1.doc]

# Supplementary Appendix 1. Literature Guidance Method

HTML5 (H5) page micro-scene is a form of network marketing which is increasingly popular with many firms and individuals. The current H5 technology can be applied to the marketing and advertising of products and services. Since the EQXIU platform provides a convenient creative platform tool for H5 page micro-scene, we aim to provide guidance on how an HUAWEI H5 micro-scene can be quickly created through the use of EQXIU.

**Step 1**: Enter <http://eqxiu.com/>, register and login into the account.

**Step 2**: Click “Create Scene” in the official website.

**Step 3**: ①Select an empty template, which can be freely used to create a micro-scene;

②Select the mode: “Create H5 micro-scene, publicity for HUAWEI”.

**Step 4**: Add and revise HUAWEI related background, photos, text and background music.

**Step 5**: The size of the photos, text, frames and animations can easily be revised by clicking on the appropriate link.

**Step 6**: Once the production has been completed, click “Publish”.

**Step 7**: Set the “Cover”, “Topic”, “Page Turning Mode” and “Description” of H5 micro scene”, and then complete the process by clicking “Save Settings”.

# Supplementary Appen**dix 2. Grap**hical Guidance Method

Similar to above, we have the following instructions for the Graphical Guidance Method group.

**Step 1**: Enter <http://eqxiu.com/>, register and login into the account.

**Step 2**: Click “Create Scene” in the official website.


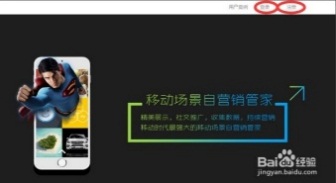

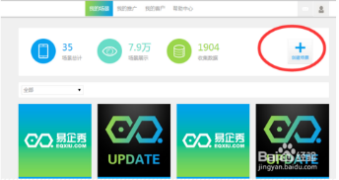


**Step 3**: ①Select an empty template, which can be freely used to create a micro-scene;

②Select the mode: “Create H5 micro-scene, publicity for HUAWEI”.

**Step 4**: Add and revise some HUAWEI related background, photos, text and background music.


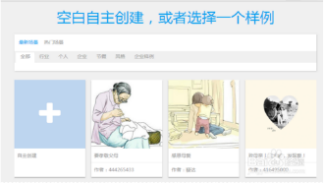

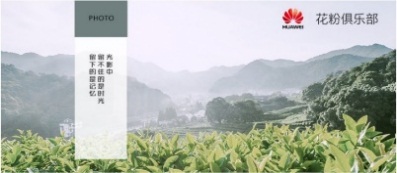


**Step 5**: The size of the photos, text, frames and animations can easily be revised by clicking on the appropriate link.

**Step 6**: Once the production has been completed, click “Publish”.


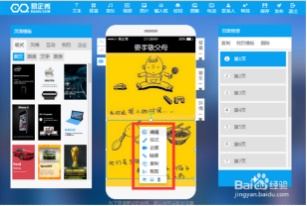

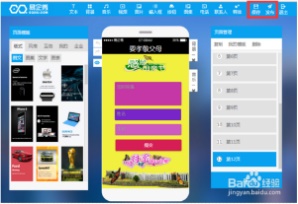


**Step 7**: Set the “Cover”, “Topic”, “Page Turning Mode” and “Description” of H5 micro-scene”, and then complete the process by clicking “Save Settings”.


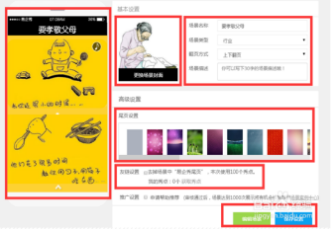


# Supplementary Appendix 3. Second Experiment No Template and Template materials

**No template materials**

**Directions:** You may freely use any pictures or music you consider suitable, but your scene must be created based on the topic of “Teacher’s Day”.

**Template materials**

**Directions:** You may freely select any of the materials made available to you (i.e., photos and music), but your scene must be created based on the topic of “Teacher’s Day”.

Music:


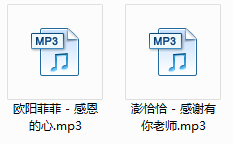


Photos:

| 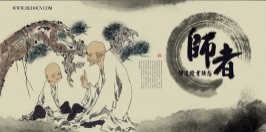 | 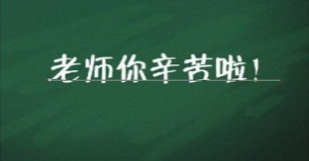 | 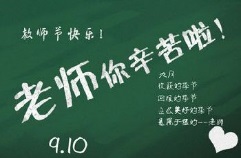 | 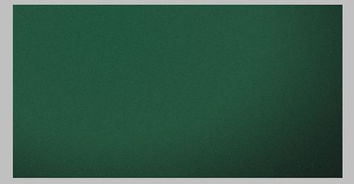 |
| --- | --- | --- | --- |
| 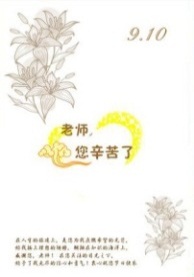 | 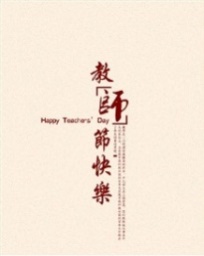 | 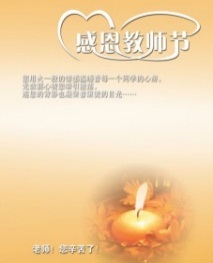 | 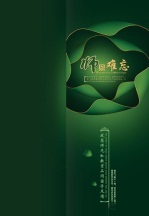 |
| 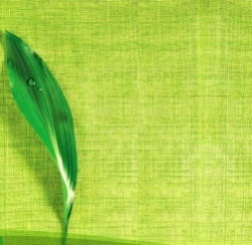 | 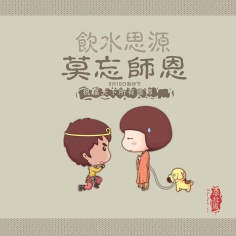 | 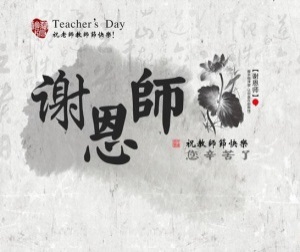 | 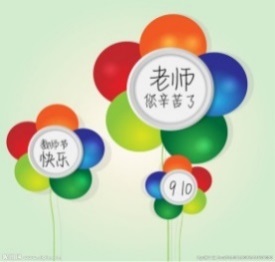 |

# Supplementary Appendix 4. Common Method Bias Analysis

**Table A1.** Testing for common method bias (First experimental design)

| Model | χ^2^ | df | Δχ^2^ | Δdf | p |
| --- | --- | --- | --- | --- | --- |
| Single factor | 539.1 | 104 | 354.9 | 3 | 0.00 |
| Multi-factor | 184.2 | 101 |

**Table A2.** Testing for common method bias (Second experimental design)

| Model | χ^2^ | df | Δχ^2^ | Δdf | p |
| --- | --- | --- | --- | --- | --- |
| Single factor | 539.7 | 104 | 317.7 | 3 | 0.00 |
| Multi-factor | 222.0 | 101 |

# Supplementary Appendix 5. Moderated Multiple Regression (PROCESS Macro Model 1)

**Table A3. Moderated multiple regression (First experimental design)**

|  | Recommendation Intentions | | | | | |
| --- | --- | --- | --- | --- | --- | --- |
|  | **Coefficient** | **SE** | ***t*** | ***p*** | **LLCI** | **ULCI** |
| Constant | 2.98 | 0.24 | 12.50 | 0.00 | 2.51 | 3.46 |
| Customer Knowledge | 0.48 | 0.15 | 3.28 | 0.00 | 0.19 | 0.78 |
| Guidance Methods | 0.76 | 0.34 | 2.21 | 0.03 | 0.08 | 1.44 |
| Customer Knowledge  × Guidance Methods | -0.39 | 0.22 | -1.82 | 0.07 | -0.83 | 0.04 |

**Table A4. Moderated multiple regression (Second experimental design)**

|  | Recommendation Intentions | | | | | |
| --- | --- | --- | --- | --- | --- | --- |
|  | **Coefficient** | **SE** | ***t*** | ***p*** | **LLCI** | **ULCI** |
| Constant | 2.28 | 0.28 | 8.11 | 0.00 | 1.72 | 2.84 |
| Customer Knowledge | 0.85 | 0.16 | 5.38 | 0.00 | 0.54 | 1.17 |
| Materials Provided | 1.19 | 0.34 | 3.49 | 0.00 | 0.51 | 1.86 |
| Customer Knowledge × Materials Provided | -0.73 | 0.20 | -3.58 | 0.00 | -1.14 | -0.33 |
